# Intravenous Lipid Emulsion for the Treatment of Perioperative Cocaine Intoxication

**DOI:** 10.7759/cureus.19146

**Published:** 2021-10-30

**Authors:** Wael Saasouh, Anuja Nikam, Bachar Hachwa

**Affiliations:** 1 Anesthesiology, Detroit Medical Center, NorthStar Anesthesia, Detroit, USA; 2 Medicine, Michigan State University, Detroit, USA

**Keywords:** emergent surgery, local anesthetic systemic toxicity, anesthesia, lipid emulsion, cocaine toxicity

## Abstract

Symptomatic cocaine intoxication in the preoperative period is a potentially life-threatening condition, especially before emergent surgery. The anesthesiologist is faced with a dilemma where the patient is deemed unsafe for induction of general anesthesia but also in need of immediate surgical intervention. Cocaine is a local anesthetic and, as such, has been proposed to respond to lipid emulsion treatment as other local anesthetics would. We present a case supporting this statement and review the relevant published literature on the topic.

## Introduction

Acutely intoxicated surgical patients present unique challenges to the anesthesiologist. Treatment of local anesthetic (LA) toxicity is multifold, and intralipid emulsion (ILE) has been advocated based on limited literature [1]. Patients with acute cocaine toxicity may require urgent treatment for tachycardia, dysrhythmia, hypertension, and coronary vasospasm in order to prevent pathological sequelae such as acute coronary syndrome, stroke, and death [2]. Cocaine inhibits catecholamine reuptake leading to cardiotoxicity [3], which may compound the clinical picture and introduce an additional boundary to effective treatment. ILE, however, has been established as a treatment for LA toxicity [4]. Cocaine being a local anesthetic, ILE has been proposed as a treatment for toxicity, although the published literature is largely heterogeneous [4-6]. We present a case where ILE was used emergently in acute cocaine toxicity.

## Case presentation

Our patient was a 37-year-old 50 kg Caucasian female with a past medical history of cerebral palsy and prior Cesarean sections admitted through the emergency department (ED) at seven weeks gestational age (WGA), with severe left lower abdominal quadrant and generalized body pain. Social history was positive for cigarette smoking, alcohol, marijuana, and cocaine use. On presentation, the patient was awake and alert with tachycardia (135 bpm, sinus on electrocardiogram), tachypnea (28 rpm), dry mucous membranes, and bilateral isolated upper extremity rest tremors were noted. She reported no angina, dyspnea, visual disturbances, severe headache, or psychological agitation. Pupils were moderately constricted and reactive. Blood pressure (BP) was 108/71. Lab work showed bicarbonate of 14 mmol/L, anion gap of 17 mmol/L, glucose 132 mmol/L, leukocytosis of 20,000 x 10^9/L, CPK of 43 units/L, hCG of 12,654 mIU/mL, blood alcohol (ethanol) level of 72 mg/dL, and a positive urine pregnancy test. Qualitative urine toxicology was positive for benzodiazepines, cannabinoids, cocaine, and opiates. Initial treatment included nil per os, 18-gauge intravenous access, fluid resuscitation with a total of three liters of saline, analgesics, and one unit packed red blood cell transfusion (point of care testing revealed an acute drop in hemoglobin to 7.1 g/dL from a baseline of 14 g/dL). Further workup revealed a ruptured ectopic pregnancy, and the patient was boarded for emergent exploratory laparotomy. Additional questioning revealed cocaine use two hours before the presentation. Given the emergent status and clinical suspicion of cocaine toxicity, she was started on an infusion of 20% ILE (Intralipid, Fresenius Kabi, Lake Zurich, Illinois, USA) with a 1.5 ml/kg loading dose (12 g) followed by an infusion of 0.5 ml/kg/min for a total of 500 ml (100 g).

In the operating room, the patient was premedicated with 0.5 mg hydromorphone and 2 mg midazolam. Induction of general anesthesia was done via 100 mcg fentanyl, 130 mg propofol, and 100 mg succinylcholine, followed by uneventful intubation, and maintenance with sevoflurane (2.1% end-tidal) and rocuronium (10 mg after the return of neuromuscular function and an additional 30 mg throughout the case). An arterial line was not available. A malignant hyperthermia emergency cart was available. Forced air warming was applied throughout. The patient received antiemetic prophylaxis (8 mg dexamethasone, 4 mg ondansetron) and two doses of 0.5 mg hydromorphone in the last 30 minutes of surgery. Total anesthesia time was around 1.5 hrs. BP was 145/75 after intubation, lowest throughout the case was 110/50 mmHg, and around 120/90 before extubation. Peripheral saturation of oxygen (SpO2) was 99%-100% intraoperatively, and the temperature was 36.7°C-36.9°C, with no additional signs of cocaine toxicity. Intraoperative electrolyte panel showed a sodium of 136 mmol/L, potassium of 4.0 mmol/L, hematocrit of 21%, and hemoglobin of 7.1 g/dL. In the post-anesthesia care unit (PACU), BP was 120/53 mmHg, heart rate 99 bpm, respiratory rate 20 rpm, and SpO2 99% on an 8 L/min simple face mask. The patient was awake and responsive.

Postoperatively, the patient was transferred to the gynecology ward. Over the following 12 hours, intravenous hydration was continued with two liters of normal saline. Her hemoglobin levels stabilized at 8 g/dL, and she was given 65 mg ferrous sulfate. The pain was managed with acetaminophen-hydrocodone (two tablets of 7.5 mg/325 mg), 30 mg of intravenous ketorolac, and hydromorphone intravenous patient-controlled analgesia (IVPCA). The patient tolerated an oral diet on the evening of surgery. She was discharged home in stable condition three days later.

## Discussion

ILE has been recommended for multiple lipid-soluble drugs, including local anesthetics [7]. Our case, along with some reported literature [6], suggests that the systemic effects of cocaine, an old local anesthetic, could potentially be reversed by applying the American Society of Regional Anesthesia and Pain Medicine protocol of intravenous lipid emulsion [8]. Prompt treatment of acute cocaine toxicity is crucial to avoid organ damage. Elevated cardiac markers, dysrhythmias, or signs of congestive heart failure may be presenting signs of early complications of acute intoxication, and overt myocardial infarction is reported in up to a third of patients [2].

The effect of ILE has been debated in cases of LA toxicity, and more specifically, cocaine overdose. Although the literature is not conclusive [1,4,5,9], there has been reported literature [7,10] of ILE rapidly improving cardiovascular parameters in the case of a cocaine overdose. The mechanism of action of ILE in LA toxicity is not fully understood, but ILE is thought to act as a “lipid sink” that provides a greater volume of distribution for passive diffusion of LA, thus decreasing its concentration in target tissues [7,11]. It has been suggested that the effect of ILE varies by the concentration of cocaine in plasma, with the “reversal” effect being more prominent at higher doses [1,6].

A more inclusive treatment plan for cocaine intoxication would have included one or more agents typically aimed at alleviating acute cardiovascular disturbances [12]. Increased sympathetic activity can be attenuated by benzodiazepines, calcium channel blockers, alpha-2 agonists, or beta-blockers. Coronary vasospasm and hypertension may be reversed with calcium channel blockers, vasodilators, alpha-blockers, alpha-2 agonists, and some beta-blockers. Some of these agents also cause decreased heart rate further, alleviating the otherwise increased myocardial oxygen consumption. Antipsychotics have been used to specifically target agitation and psychosis. Several other agents are not well-studied (including anesthetic medications and ILE), and no clear guidelines could be drawn regarding their utility. Our patient only received a fraction of this list of medications as her symptoms and vital signs seemed to significantly improve after the ILE administration. It is worth considering that every additional medication introduces the potential for adverse events, however, patient interview in the ED did not elicit some of the other signs of cocaine toxicity, such as acute altered mental status, focal neurological signs (seizure, confusion, or motor weakness), acute chest pain, epistaxis, psychological disturbances, or vascular spasms [3]. Some signs could have been attributed to her ruptured ectopic pregnancy such as abdominal pain, nausea, vomiting, and diaphoresis, among others. While we had no means of definitively confirming cocaine toxicity in our patient, the suggestive history, lifestyle risk factors, and subsequent response to ILE make this theory highly plausible. The leukocytosis, hypertension, low-grade fever, and tachycardia observed before ILE administration may have been ectopic, but the short-term response to ILE administration makes that unlikely.

Our patient’s presentation and response to treatment suggest that the ILE may have played a pivotal role perioperatively. The adverse effects of ILE administration, such as organ damage, hypersensitivity, and cardiovascular events, must be kept in mind, and definitive evidence is required before recommendations can be made regarding this treatment modality [13]. As the literature stands, ILE may be used to reverse systemic effects of acute cocaine toxicity when other therapies are lacking or ineffective [4].

## Conclusions

Lipid emulsion treatment of acute cocaine toxicity may potentially be utilized in the perioperative or emergency settings. Definitive data regarding this modality is lacking, but current published evidence suggests it could be a viable option, especially when other measures have failed.
